# Infective endocarditis on mitral annular calcification: a case report

**DOI:** 10.1186/1757-1626-2-9072

**Published:** 2009-11-20

**Authors:** Giovanni Minardi, Paolo G Pino, Martina Sordi, Herribert Pavaci, Carla Manzara, Giovanni Pulignano, Enrico Natale, Carlo Gaudio

**Affiliations:** 1 Cardiodiagnostica non invasiva, Department of Cardiology and Cardiovascular Surgery, Azienda Ospedaliera San Camillo-Forlanini, Rome, Italy; 2 Second Division of Cardiology, Department of Heart and Great Vessels Attilio Reale, "Sapienza", University of Rome, Rome, Italy

## Abstract

**Introduction:**

Mitral annular calcification is a common finding in elderly patients; it is considered a benign cardiac abnormality but it can be a predisposing factor for infective endocarditis. Although described in numerous necropsy studies, endocarditis on mitral annular calcification has rarely been reported during life, and the frequency of sepsis can be underestimated because of difficult diagnosis.

**Case presentation:**

We present a case of infective endocarditis on mitral annulus calcification in a patient with acute coronary syndrome, diagnosed with transthoracic echocardiography.

**Conclusion:**

Transthoracic echocardiography may contribute to a correct diagnosis, showing typical findings of infective endocarditis on mitral annular calcification in order to administrate an adequate antibiotic prophylaxis in patients undergoing endoscopic or invasive procedures.

## Introduction

Mitral annular calcification (MAC) is a chronic degenerative process, which occurs mainly in elderly patients and more frequently in those with chronic renal failure receiving long-term haemodialysis. MAC is considered to be the result of a degenerative process within the cardiovascular fibrous skeleton. In younger patients, the conditions can be seen in association with factors that accelerate this process, including connective tissue diseases, metabolic disorders or conditions that increase mitral annular stress [1-3]. Although it is considered a benign cardiac abnormality, it can be a predisposing factor for infective endocarditis. Infective endocarditis (IE) on MAC is described in numerous necropsy studies, but has rarely been reported during life [4].

In this report, we describe a patient with a calcification of the mitral annulus complicated by infective endocarditis.

## Case presentation

A 78-year-old italian man was referred to our hospital because of oppressive chest pain. His past history included myocardial infarction and cerebral transient ischemic attach (TIA), hypercholesterolemia, systemic hypertension, chronic renal failure, peripheral arterial disease and anemia; he had also marked limitation of physical activity. At the admission the electrocardiogram (ECG) showed a right bundle branch block, left anterior hemiblock, Q waves in anterior and inferior leads and ST abnormalities in anterior leads; myoglobin was 228 ng/ml and troponin 12 ng/ml. So diagnosis was ACS-NSTEMI. Antiaggregant and anticoagulant treatment, including glycoprotein IIb/IIIa inhibitors, was administered; because of urination difficulty, a bladder catheter was inserted. Congestive heart failure was documented with impaired functional class (NYHA III-IV).

Seven days after recovery, the patient became tachypnoic and feverish. His body temperature was 38.4°C, blood pressure was 150/70 mmHg and pulse rate 90 beats/min. Physical examination revealed a pansystolic murmur of grade 3/6 audible on mitral area and there were signs of peripheral embolism (Osler noduli). Laboratory examinations showed an hypocromic and microcytic anemia (with hemoglobin 8.8 g/dl and hematocrit 27.7%), polymorphonuclear leukocytosis (white blood cells 17,7 × 10^3^/ml). Other findings were as follow: glycaemia 134 mg/dl, urea 174 mg/dl, creatinine 2.93 mg/dl, phosphate 3.64 mmol/L and myoglobin 343 ng/ml. Urine cultures resulted positive for *Klebsiella pneumoniae*.

**Figure 1:**
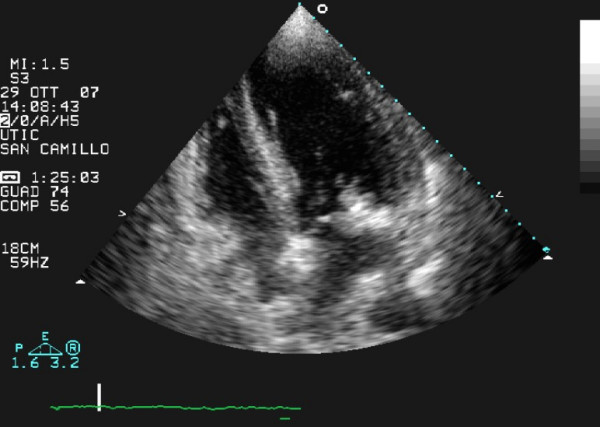
**Two-dimensional echocardiogram, apical 4-chamber view: vegetation is seen attached to calcified mitral annulus**.

**Figure 2:**
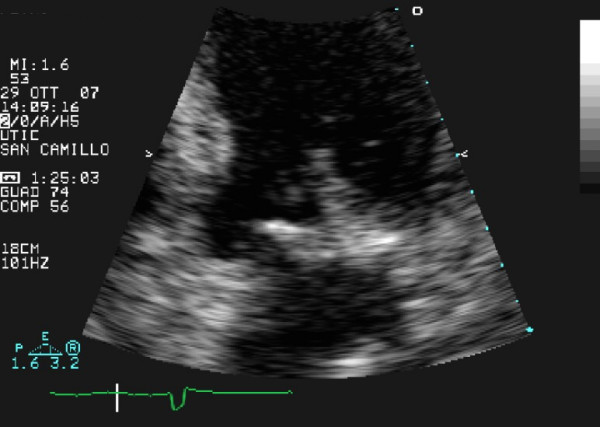
**Two-dimensional echocardiogram, apical 4-chamber view (detail): vegetation is shown at a higher magnification**.

Transthoracic echocardiography (TTE) revealed a calcified mitral annulus with an echo-dense spherical, tumor-like mass, located close to the posterior leaflet (Figure 1). A typical vegetation was located at the base of the posterior mitral leaflet, especially on the ventricular surface (Figure 2). These findings confirmed the diagnosis of IE associated with MAC. On Doppler color-flow mapping, a mild mitral regurgitation was seen in the left atrium; no obstruction to the diastolic transmitral flow was found. The left ventricle was dilated with akinesia of the apex and hypokinesia of the other segments and an ejection fraction of 30%. The right ventricle and right atrium were normal. The aortic valve was tricuspid and showed some calcification.

Blood cultures were performed three times, and methicillin-resistant *Staphylococcus aureus *(MRSA) grew in all samples. An antibiotic therapy was started with rifampicin 600 mg/die and oxacillin 8 g/die.

The patient died few days later for multiorgan failure.

## Discussion

Calcification of the mitral annulus is a fairly common finding in adults at autopsy and it is more than twice as frequent in women (11.5%) as in men (4.5%) and the frequency rise sharply with increasing age. The cause of the calcification is not known, but a degenerative change in the connective tissue of the annulus is favoured [5].

Although it is usually considered a benign finding, it has been shown to be associated with various pathologic conditions such as aortic stenosis, hypertrophic cardiomyopathy, hypertension, mitral valve prolapse, diffuse conduction system disease or arterial embolization [3]. Some of these conditions may contribute to calcification. One complication of MAC is bacterial endocarditis [6] and sepsis has been reported as a rare and severe complication. Several reports have been made concerning IE of the MAC, the majority of them based on autopsy studies [7]. A prospective study [8] of 976 elderly patients, including 526 patients with MAC, demonstrated at 39-month mean follow-up a higher incidence of bacterial endocarditis in patients with MAC (3%) than in patients without MAC (1%). One study [9] made clinical and echocardiographic investigation about IE and MAC, studying 62 cases of IE of the mitral valve over a five years period with transesophageal echocardiography: among these patients, 15 (24%) had vegetations originating from a MAC, while 47 had classic leaflet endocarditis: among these patients, those with IE on MAC had a higher incidence of diabetes mellitus and cancer.

Methicillin-resistant *Staphylococcus aureus *(MRSA) is the most frequent infective agent [10]. MAC may predispose to infection determining a turbulent flow and traumatizing the endothelium: so this provides a good field to bacterial nidation, because endothelial erosion exposes the calcific mass to transient bacteraemia [7]. It's difficult to prove this specific diagnosis in vivo because of the scarcity of signs. TTE probably usually misses the vegetation because of poor imaging conditions and calcification generates artefacts.

The pertinent clinical findings in our study were, in an elderly patient with ACS-NSTEMI and MAC, the appearance of a murmur of mitral insufficiency, fever, urinary infection, anemia, polymorphonuclear leukocytosis, three positive blood cultures, systemic embolism and TTE findings of IE. Therefore, we believe that the calcification of the mitral valve annulus must be defined in patients over 60 years of age, and, although a potential risk has not been clearly established, that the antibiotic prophylaxis could be recommended before digestive or urological endoscopic procedures and in those conditions in which the risk of IE become significantly high (hospitalization, bladder catheterization, central venous catheter).

## Conclusion

The diagnosis of IE should be considered in any elderly patient who has a calcified mitral annulus, in which clinical and laboratory findings suggestive of IE occur. TTE may contribute to a correct diagnosis, showing typical findings of IE on MAC. Transesophageal echocardiography may be useful in patients with non-diagnostic TTE. We recommend to administer an adequate antibiotic prophylaxis in patients with MAC undergoing endoscopic or invasive procedures.

## Consent

Written informed consent was obtained from the patient's family for publication of this case report and accompanying images. A copy of the written consent is available for review by the Editor-in-Chief of this journal.

## Competing interests

The authors declare that they have no competing interests.

## Authors' contributions

GM and PGP made substantial contributions to analyze and interpret the patient data; CM and GP performed TTE; GM, HP, MS and CG have been involved in drafting the manuscript and revising it critically; all authors have given final approval of the version to be published.
